# Wisteria Floribunda Agglutinin-Labeled Perineuronal Nets in the Mouse Inferior Colliculus, Thalamic Reticular Nucleus and Auditory Cortex

**DOI:** 10.3390/brainsci6020013

**Published:** 2016-04-13

**Authors:** Sarah M. Fader, Kazuo Imaizumi, Yuchio Yanagawa, Charles C. Lee

**Affiliations:** 1 Department of Comparative Biomedical Sciences, School of Veterinary Medicine, Louisiana State University, Baton Rouge, LA 70803, USA; smcrey1@lsu.edu; 2 Stanley Center for Psychiatric Research, Broad Institute of MIT and Harvard, Cambridge, MA 02142, USA; imaizumi@broadinstitute.org; 3 McGovern Institute for Brain Research, MIT, Cambridge, MA 02139, USA; 4 Department of Genetic and Behavioral Neuroscience, Gunma University Graduate School of Medicine, Maebashi, Gunma 371-8510, Japan; yuchio@gunma-u.ac.jp

**Keywords:** auditory, thalamus, cortex, midbrain, thalamic reticular nucleus, inferior colliculus, auditory cortex, wisteria floribunda agglutinin

## Abstract

Perineuronal nets (PNNs) are specialized extracellular matrix molecules that are associated with the closing of the critical period, among other functions. In the adult brain, PNNs surround specific types of neurons, however the expression of PNNs in the auditory system of the mouse, particularly at the level of the midbrain and forebrain, has not been fully described. In addition, the association of PNNs with excitatory and inhibitory cell types in these structures remains unknown. Therefore, we sought to investigate the expression of PNNs in the inferior colliculus (IC), thalamic reticular nucleus (TRN) and primary auditory cortex (A1) of the mouse brain by labeling with wisteria floribunda agglutinin (WFA). To aid in the identification of inhibitory neurons in these structures, we employed the vesicular GABA transporter (VGAT)-Venus transgenic mouse strain, which robustly expresses an enhanced yellow-fluorescent protein (Venus) natively in nearly all gamma-amino butyric acid (GABA)-ergic inhibitory neurons, thus enabling a rapid and unambiguous assessment of inhibitory neurons throughout the nervous system. Our results demonstrate that PNNs are expressed throughout the auditory midbrain and forebrain, but vary in their local distribution. PNNs are most dense in the TRN and least dense in A1. Furthermore, PNNs are preferentially associated with inhibitory neurons in A1 and the TRN, but not in the IC of the mouse. These data suggest regionally specific roles for PNNs in auditory information processing.

## 1. Introduction

The processing of sensory information in the brain relies on the development of an intricate network of connections linking widespread neural structures [1,2,3]. The regulation of synaptic transmission at these sites is regulated both at the neuronal synaptic junction and by supporting elements, such as glial cells [4] and collections of extracellular matrix proteins, broadly termed perineuronal nets (PNNs) [5]. The latter, in particular, is beginning to see renewed interest, particularly in the auditory nervous system, as reviewed in Sonntag *et al.* (2015). These PNNs are particularly interesting within the context of information flow though the auditory system, due to their putative functional roles in mediating synaptic transmission and plasticity [5,6,7,8,9]. As such, PNNs have been characterized neuroanatomically in most of the major auditory structures of common lab animals [10,11,12,13,14,15,16,17,18,19,20]. Interestingly though, although PNNs have been described and examined developmentally in the central nervous system of the mouse [14,17,21], their expression in higher auditory structures of the mouse has not been fully described.

In particular, the expression of PNNs in the auditory mid- and forebrain of the mouse is of interest, given their essential roles in the higher order processing of auditory information [22,23,24,25]. The inferior colliculus (IC) is a major hub for both ascending, descending and lateral information [22,26,27], while the thalamic reticular nucleus (TRN) is the principal source of inhibition to the auditory thalamus, medial geniculate body (MGB), in mice and other rodents, where inhibitory neurons are nearly non-existent [28,29] and where no WFA-labeled PNNs were found in our study. The primary auditory cortex (A1) is the upstream target for ascending auditory information [23,30,31,32]. Consequently, characterizing the degree of expression and distribution of PNNs in these central auditory neural structures of the mouse will further enable defining their distinct roles in auditory information processing. Moreover, we have previously focused our attention on auditory processing in these regions [25,33] and thus chose to specifically focus on those regions here, since PNNs in some lower auditory brainstem nuclei in the mouse are well investigated anatomically and physiologically [5,17].

Therefore, in this study, we examined the expression and distribution of PNNs, by characterizing their reactivity with wisteria floribunda agglutinin (WFA) [34,35]. Moreover, we utilized a transgenic mouse line that expressed the Venus-fluorescent protein in presumed inhibitory neurons of these auditory structures, *i.e.*, the vesicular GABA transporter (VGAT)-Venus transgenic mouse line [36,37], which we have employed in previous studies of the auditory system [26,38]. This mouse line is particularly useful for the expeditious identification of gamma-amino butyric acid (GABA)-ergic inhibitory neurons throughout the nervous system, since it expresses an enhanced form of yellow-fluorescent protein natively in nearly all GABAergic inhibitory neurons [36,37]. We found that PNN expression in the IC, TRN, and AI varied both within and between structures, as discussed below.

## 2. Methods

### 2.1. Histology

The Institutional Animal Care and Use Committee of the Louisiana State University School of Veterinary Medicine approved all of these procedures. To examine the distribution of PNNs relative to inhibitory neuronal populations, we used a transgenic mouse strain that expresses the Venus-fluorescent protein in neurons expressing the vesicular gamma-amino butyric acid transporter (VGAT). These VGAT-Venus mice were bred on a C57BL/6 background and ranged in ages from 3–9 months (3 months: *n* = 8; 6 months: *n* = 6; 9 months: *n* = 6). A notable caveat to these studies is that, like most transgenic mice strains, the VGAT-Venus strain was created and bred on the C57BL/6 background [36,37], which is known to have an early degeneration of the stria vascularis, resulting in early and progressive hair cell loss in the cochlea [39,40]. Thus, as noted, we compared results across different age groups to assess whether PNN formation potentially also varied with age in this mouse strain. The Venus fluorescent protein was developed by Atsushi Miyawaki at RIKEN, Wako, Japan and the VGAT-Venus transgenic mouse was developed and shared by permission from Yuchio Yanagawa at Gunma University and obtained from Janice R. Naegele at Wesleyan University [36,37].

To collect brains for histological analysis, mice were first anesthetized using inhalation of isoflurane anesthesia in an enclosed chamber until reflex responses were no longer present. Transcardiac perfusion was used to fix the brain with a solution containing 4% paraformaldehyde (Electron Microscopy Sciences, Hatfield, PA, USA) in 0.01 M phosphate buffered saline (PBS) at pH 7.4. Brains were then post-fixed overnight in the 4% paraformaldehyde solution and then transferred the following day to a 4% paraformaldehyde/30% sucrose solution in 0.01 M PBS for 2–3 days for cryoprotection. The brains were blocked using a clean razor blade to preserve the structures of interest, either coronally for the IC or in the thalamocortical slice plane for A1 and TRN [41]. The thalamocortical plane of section is an off-horizontal sectioning plane developed by Cruickshank *et al.* (2002) that preserves intact connections between the MGB and A1 in acute *in vitro* slice preparations, which we also employ in our histological studies for comparison with our related physiological investigations [32,42,43]. It also enables the simultaneous investigation of several relevant central auditory structures. The blocked brains were then mounted on a stage for cryostat sectioning at 50 μm (Leica Microsystems, Buffalo Grove, IL, USA). Individual brain sections were then collected in 0.01 M PBS and transferred to 48 well plates for processing.

To visualize PNNs, sections were washed in 0.01 M PBS and then permeablized in 0.3% Triton X-100 in 0.01 M PBS for 1 h. These sections were first counterstained using To-Pro-3 (Thermo Fisher Scientific, Waltham, MA, USA) and washed in 0.01 M PBS twice. Then, the sections were blocked by streptavidin solution and biotin solution (Vector Labs, Burlingame, CA, USA), separately, for 15 min, washed in 0.01 M PBS, and incubated in a solution containing biotinylated wisteria floribunda agglutinin (WFA) (Vector Labs, Burlingame, CA, USA) overnight at room temperature. Sections were then washed in 0.01 M PBS before being transferred to Alexa568 streptavidin (Thermo Fisher Scientific, Waltham, MA, USA) for 30 min at room temperature. After rinsing in 0.01 M PBS, the sections were mounted on gelatinized slides and coverslipped with VectaShield Mounting medium (Vector Labs, Burlingame, CA, USA).

### 2.2. Analysis

Slides were visualized on a Leica TC SP2 confocal microscope housed in the departmental microscopy facility or an Olympus BX-51 epifluorescence microscope. Adjacent images from each section were acquired at 10X magnification and photomontaged using Adobe Photoshop (Adobe Systems, San Jose, CA, USA). Cytoarchitectonic and gross anatomical features determined the laminar, areal, and nuclear borders in each section. In each case, three non-adjacent sections from each region (A1, TRN, IC) were selected for analysis randomly and measurements of the VGAT-positive neuron area and density and the WFA-labeled PNN distribution and density. These quantitative analyses were performed on photomontaged images of these regions using the measurement tools in Image J (NIH, Bethesda, MD, USA) and were performed by an investigator blinded to the source of each image. For each animal, we counted on average for the total of three sections in the IC (VGAT neurons: *n* = 2338; PNNs: *n* = 774), TRN (VGAT neurons: *n* = 2093; PNNs: *n* = 1003), and for A1 (VGAT neurons: *n* = 2429; PNNs: *n* = 82). Data was compiled using Excel (Microsoft, Redmond, WA, USA) and statistical analysis performed using Prism (GraphPad Software, La Jolla, CA, USA). Results were analyzed by age (3, 6, and 9 months) and as a group to assess any potential aging-related alterations to PNNs in these auditory structures.

## 3. Results

**Figure 1:**
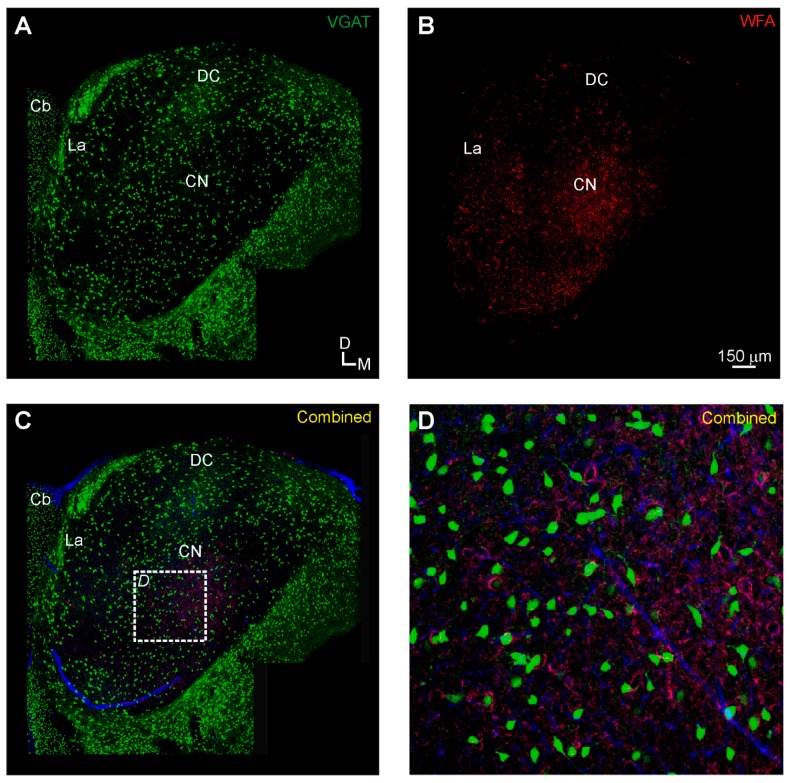
Inferior colliculus (IC) expression of Venus-positive neurons and WFA-labeling in the vesicular gamma-amino butyric acid transporter (VGAT)-Venus C57BL/6 mouse strain: (**A**) Venus-positive neurons (green) are found throughout the IC, with modular clusters in the lateral nucleus (La); (**B**) perineuronal net (PNN) expression (red) is particularly dense in the central nucleus of the inferior colliculus; and (**C**–**D**) VGAT-Venus positive neurons are often found surrounded by perineuronal nets. Panel D is a higher magnification image of the boxed region in C. Cb, cerebellum; CN, central nucleus of the IC; DC, dorsal cortex of the IC; La, lateral nucleus of the IC.

**Figure 2:**
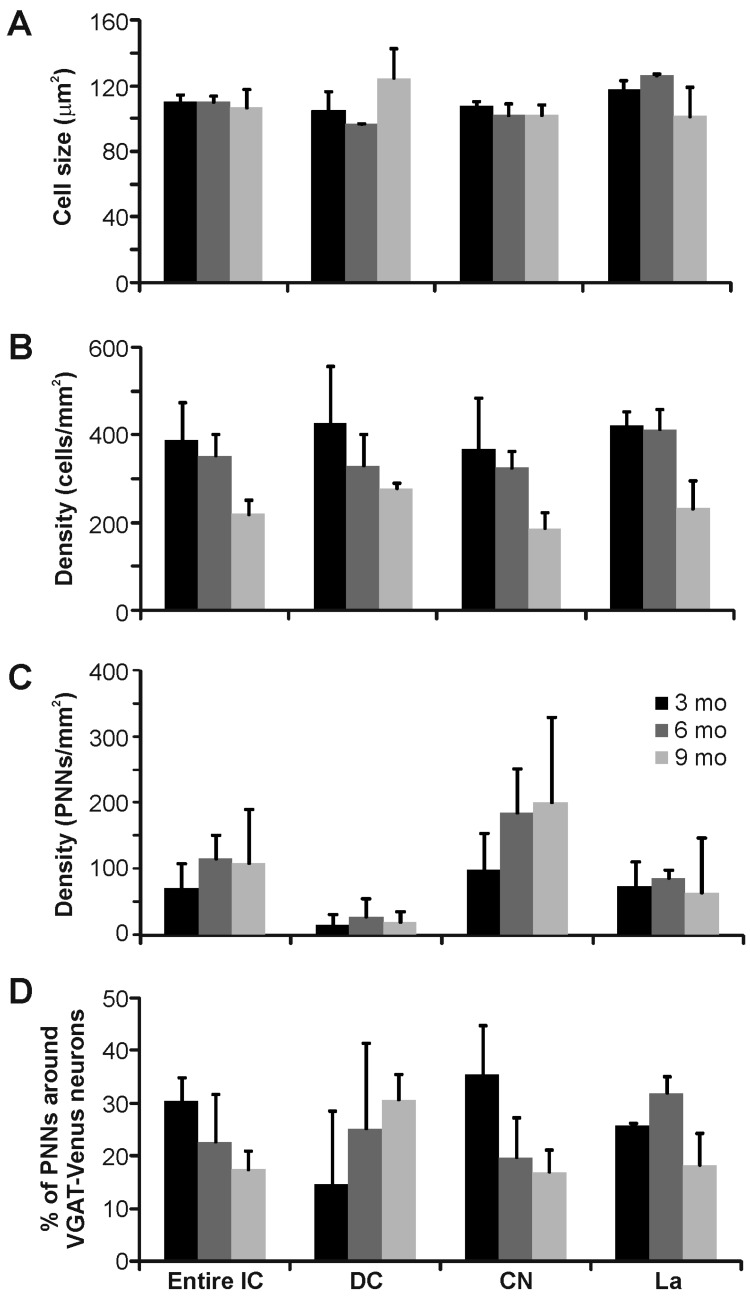
Quantification of Venus-positive and WFA-positive labeling in the inferior colliculus of the vesicular gamma-amino butyric acid transporter (VGAT)-Venus C57BL/6 mouse strain: (**A**) average size of Venus-positive neurons was similar across nuclei; (**B**) density of Venus-positive neurons was less in older animals; (**C**) PNNs were denser in the central nucleus (CN) compared with the dorsal cortex (DC) or lateral nucleus (La); and (**D**) fewer than half of PNNs surrounded VGAT-Venus positive neurons in the IC.

**Figure 3:**
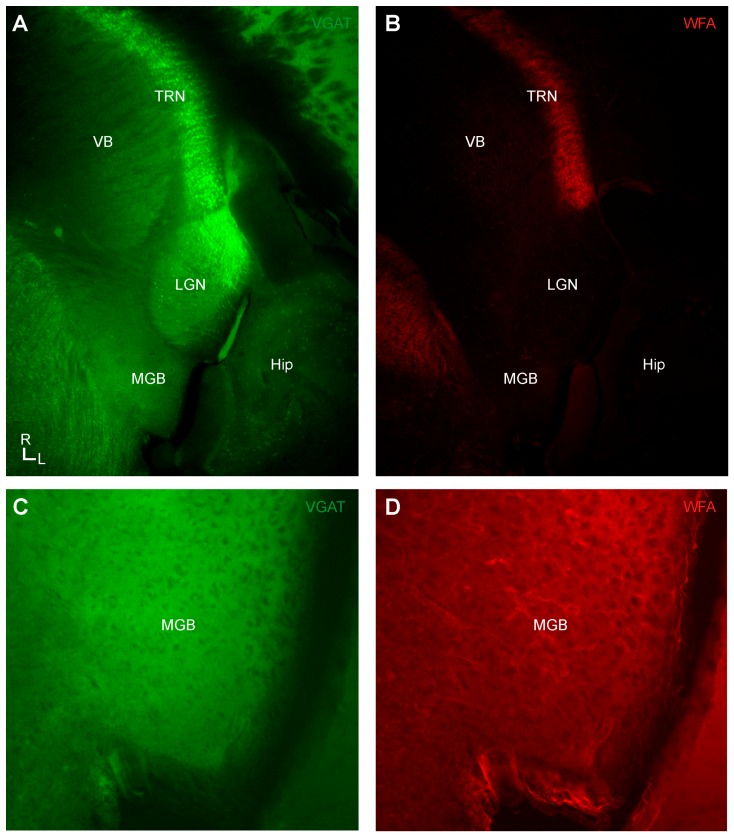
In the vesicular gamma-amino butyric acid transporter (VGAT)-Venus C57BL/6 mouse strain, wisteria floribunda agglutinin (WFA)-labeled perineuronal nets (PNNs) are absent in the medial geniculate body (MGB), ventrobasal complex (VB), and lateral geniculate nucleus (LGN), although abundant in the thalamic reticular nucleus (TRN): (**A**) epifluorescent image of VGAT-positive neurons (green) in each of the main sensory thalamic nuclei (MGB, VB, LGN) and the TRN; (**B**) epifluorescent image of WFA-labeled PNNs (red) in the sensory thalamic nuclei (MGB, VB, LGN) and the TRN; and (**C**–**D**) higher-magnification image of the MGB from A–B.

**Figure 4:**
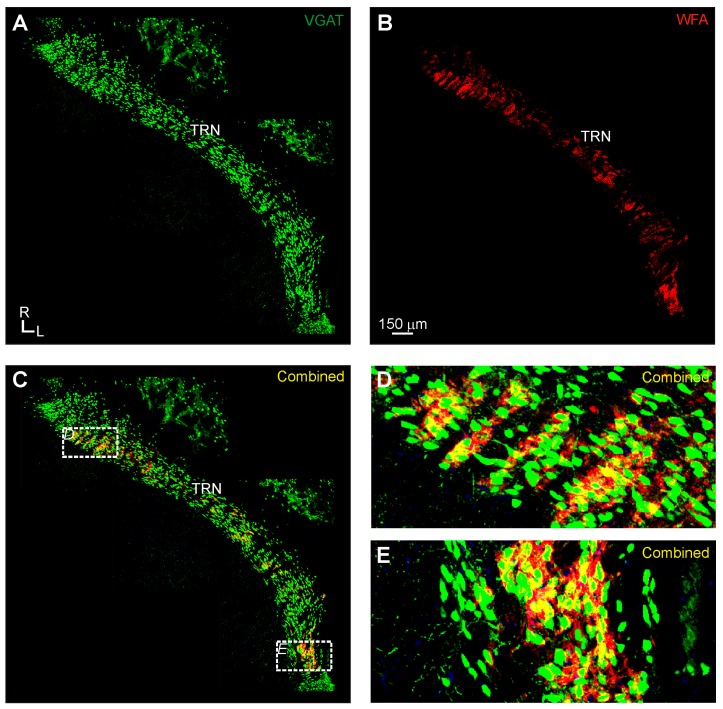
Perineuronal net (PNN) expression in the thalamic reticular nucleus (TRN) of the vesicular gamma-amino butyric acid transporeter (VGAT)-Venus C57BL/6 mouse strain: (**A**) the distribution of VGAT-positive neurons (green) was non-homogeneous across different portions of the TRN; (**B**) PNNs (red) were found highly clustered, forming banded regions in lateral and medial locations of the TRN, which was the main region in the thalamus where robust perineuronal net expression was found, for comparison see the medial geniculate body (MGB), lateral geniculate nucleus (LGN), and ventrobasal nuclei (VB) in Figure 3B for an overview of the paucity of wisteria floribunda agglutin (WFA) labeled perineuronal nets in these nuclei; (**C**) although all PNNs found surrounded Venus-positive cell bodies, not all Venus-positive cell bodies were surrounded by PNNs; and (**D**) higher-magnification view of boxed regions in panel C.

**Figure 5:**
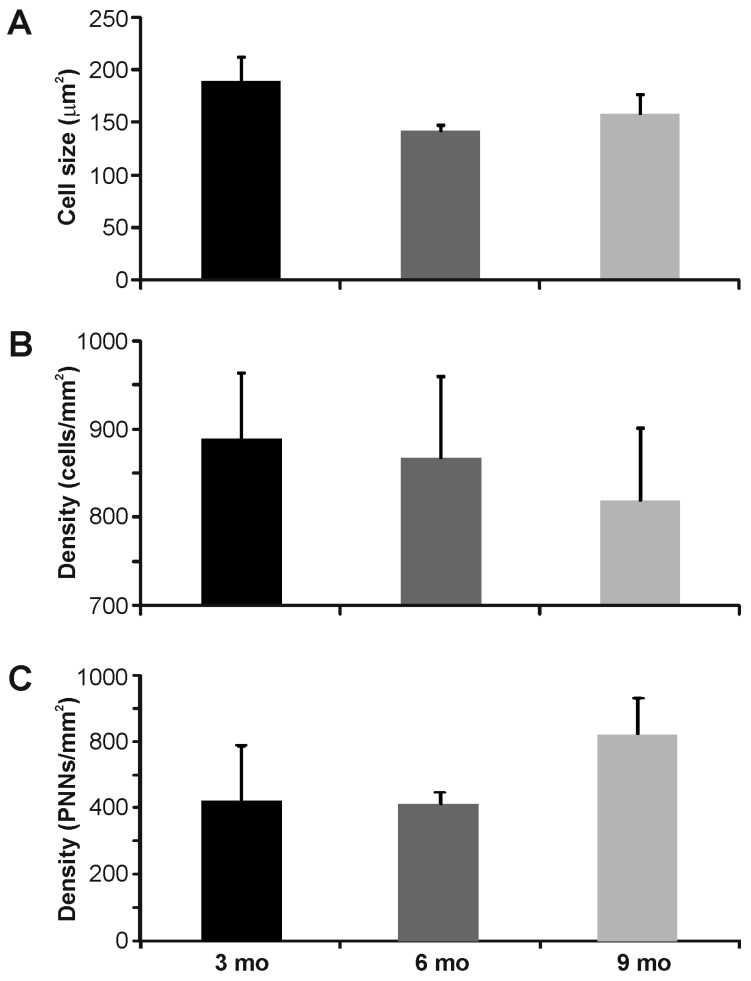
Quantification of size and density of Venus and wisteria floribunda agglutinin (WFA) labeling in the thalamic reticular nucleus (TRN) of the vesicular gamma-amino butyric acid transporeter (VGAT)-Venus C57BL/6 mouse strain: (**A**) size of Venus-positive cell bodies in three different age groups; (**B**) density of Venus-positive cells throughout the TRN; and (**C**) density of perineuronal nets (PNNs) across the TRN. mo: months.

**Figure 6:**
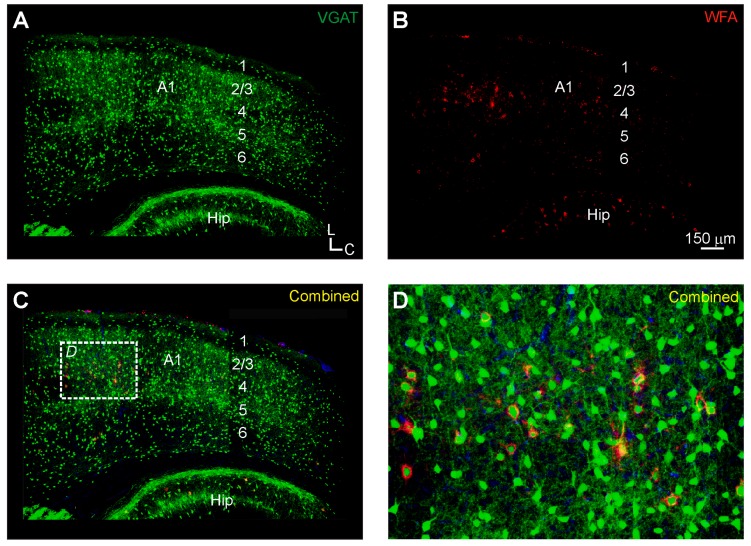
Perineuronal net (PNN) expression in the primary auditory cortex (A1) of vesicular gamma-amino butyric acid transporter (VGAT)-Venus C57BL/6 mice: (**A**) VGAT-Venus positive cell bodies and fibers (green) are found throughout all layers of the auditory cortex; (**B**) wisteria floribunda agglutinin (WFA)-labeled perineuronal nets (PNNs) were found scattered throughout the auditory cortex (A1), but were particularly dense in layer 4; and (**C**–**D**) these PNNs often surrounded the VGAT-Venus positive cell bodies, and appeared to be more highly expressed in the rostral portions of the primary auditory cortex. Panel D is magnified from boxed region in Panel C. A1, primary auditory cortex; Hip, hippocampus; 1–6, cortical layers 1–6.

**Figure 7:**
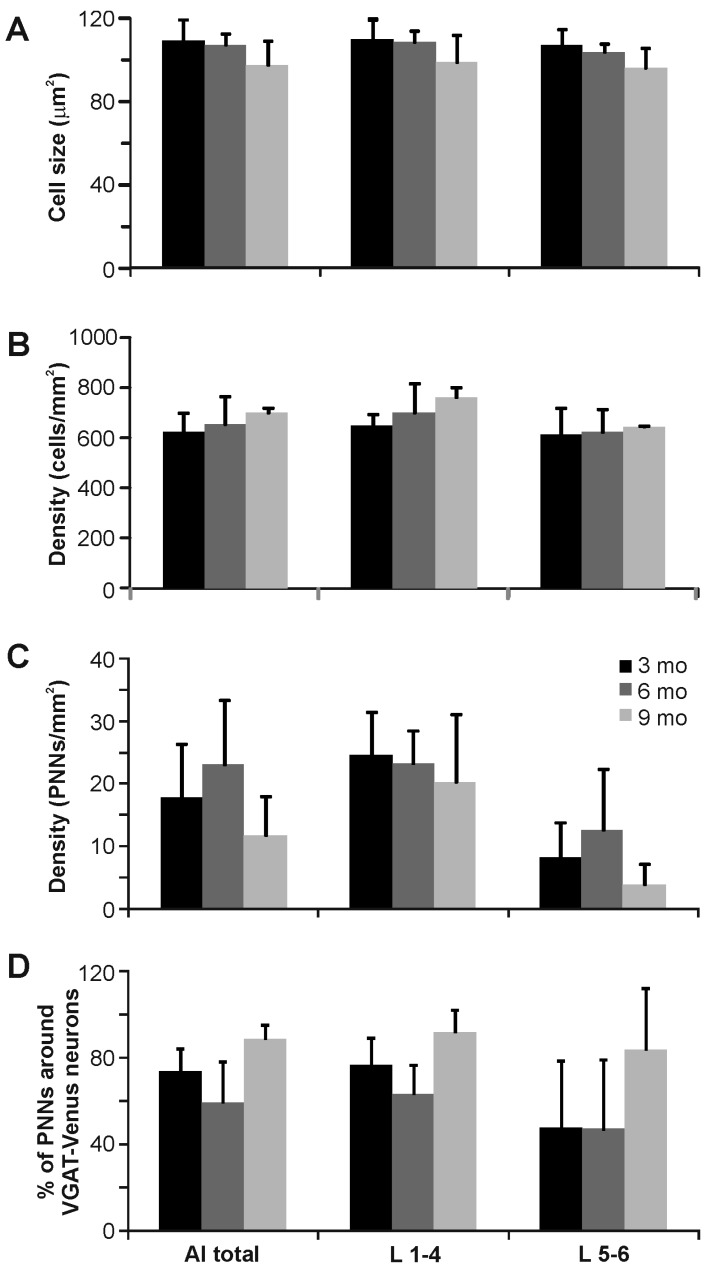
Quantification of vesicular gamma-amino butyric acid transporter (VGAT)-Venus neurons and perineuronal nets (PNNs) in the auditory cortex by layer and age in the VGAT-Venus C57BL/6 mouse strain: (**A**) average cell size (μm^2^) of VGAT-Venus positive neurons was similar across layers and areas; (**B**) density of VGAT-Venus positive cell bodies was an order of magnitude greater than perineuronal net density; (**C**) density of PNNs was greater in upper layers (L1–4) than lower layers (L5–6) of auditory cortex; and (**D**) more than half of all PNNs were found surrounding Venus-positive cells.

We examined the expression of perineuronal nets (PNNs) in the brains of vesicular gamma-amino butyric acid transporter (VGAT)-Venus C57BL/6 transgenic mouse through labeling with wisteria floribunda agglutinin (WFA), focusing on the inferior colliculus (IC), thalamic reticular nucleus (TRN), and primary auditory cortex (A1). The organization of WFA-labeled PNNs relative to presumed inhibitory neurons in these structures was assessed by examining their expression relative to the Venus-fluorescent protein, which was selectively expressed in VGAT-positive neuronal cell bodies [36]. Overall, PNN expression was evident in all of these structures, except for the MGB, often associated with presumed inhibitory neurons, (Figure 1, Figure 2, Figure 3, Figure 4, Figure 5, Figure 6 and Figure 7). As a group, PNNs were most densely expressed in the TRN (Figure 5C) and least densely expressed in the auditory cortex (Figure 7C), although their distribution was non-homogeneous in the structures examined (Figure 1, Figure 2, Figure 3, Figure 4, Figure 5, Figure 6 and Figure 7).

In the inferior colliculus, we observed WFA-labeled PNNs in all of the main subdivisions of the IC. PNNs were most densely expressed in the central nucleus of the IC (160.9 ± 40.0 PNNs/mm^2^) and least dense in the dorsal cortex of the IC (19.4 ± 6.4 PNNs/mm^2^), with the lateral nucleus intermediate in expression (73.6 ± 35.9 PNNs/mm^2^), but did not vary significantly with age (*p* > 0.05, ANOVA) (Figure 1B–D and Figure 2C). Roughly one-quarter of these WFA-labeled PNNs were associated with presumed inhibitory (VGAT-Venus positive) neurons (23.4% ± 2.9%), which did not differ significantly among each nuclei: dorsal cortex (23.4% ± 6.1%), central nucleus (24.0% ± 2.6%), lateral nucleus (25.5% ± 2.9%) (Figure 1C,D and Figure 2D). Even the densely VGAT-Venus positive modules in the lateral nucleus did not exhibit an increased proportion of WFA-labeled PNNs (Figure 5C) [44,45].

In the thalamus, no WFA-labeled PNN-labeling was observed in the principal auditory thalamic nucleus, the medial geniculate body (Figure 3), nor were any present in the surrounding visual (lateral geniculate nucleus: LGN) or somatosensory thalamic nuclei (ventrobasal nuclei: VB). This was consonant with the general paucity of VGAT-positive neurons in the MGB and VB, which instead are mainly innervated by inhibitory projections from the TRN (Figure 4 and Figure 5).

In the thalamic reticular nucleus, WFA-labeled PNNs were found throughout the lateral to medial extent of the TRN at the dorsoventral level of the medial geniculate body and auditory cortex in the thalamocortical slice plane that we employed (Figure 4). These WFA-labeled PNNs formed banded regions of intense expression, interspersed with regions devoid of expression (Figure 4B). This was evident particularly in the lateral and medial portions of the TRN (Figure 4C–E). This banding mimicked, but was not completely aligned with, similar structural banding of VGAT-Venus positive neurons in the TRN (Figure 4A). As expected, VGAT-Venus expression was ubiquitous in the TRN, which is a main source of inhibitory inputs to the medial geniculate body (Figure 4A and Figure 5A,B) and all WFA-labeled perineuronal nets (100%) were found surrounding these VGAT-Venus positive cell bodies (Figure 4C–E).

Finally, in the primary auditory cortex, WFA-labeled PNNs were found in all cortical layers, although they were most densely expressed in the upper cortical layers, particularly in layer 4 (Figure 6B–D and Figure 7C). In the lower layers, layer 6 was more intensely labeled than layer 5 (Figure 6B,C). WFA-labeled PNNs were localized towards more rostral regions of the primary auditory cortex, which may be related to the tonotopic organization of higher frequencies in this region of A1 (Figure 6D). We also assessed whether the WFA-labeled PNNs were preferentially expressed around presumed inhibitory neurons in the VGAT-Venus transgenic mouse strain (Figure 6A and Figure 7A,B). Overall, roughly three-quarters of all WFA-labeled PNNs in the cortex (73.6% ± 14.7%) were found to be associated around VGAT-Venus positive cell bodies and was not statistically different for both upper (76.9% ± 14.3%) and lower (59.3% ± 20.8%) cortical layers, nor for different ages (*p* > 0.05, ANOVA) (Figure 7D). The general paucity of VGAT-positive neurons with WFA-labeled PNNs may be indicative of the potential association of PNNs with parvalbumin-positive cells [46] which are a subset of the total inhibitory neuronal population in the brain.

## 4. Discussion

In our study, we used wisteria floribunda agglutinin (WFA) labeling to assay the expression of perineuronal nets (PNNs) in the central auditory system of the mouse, particularly focusing on the primary auditory cortex (A1: Figure 1 and Figure 2), thalamic reticular nucleus (TRN: Figure 4 and Figure 5), and the inferior colliculus (IC: Figure 6 and Figure 7). We assessed the expression of PNNs detected in this manner relative to presumed inhibitory neuronal cells in the vesicular GABA transporter (VGAT)-Venus transgenic mouse line, which expresses the Venus fluorescent protein, an enhanced form of YFP, in VGAT-positive neurons [36,37]. Overall, we found that WFA-labeled PNNs were expressed in all of these regions, but most densely in the TRN and A1, and were highly associated with inhibitory neurons in the TRN and A1, but not the IC.

A methodological caveat to our findings is our use of WFA for identifying PNNs. The composition of PNN subcomponents is thought to vary among brain regions and species [6,21,34], with the putative unit detected by WFA situated on side chains of the CSPG aggrecan component [34]. An alternative method for detection is through the use of PNN specific antibodies, however even those do not detect all PNNs [47]. Consequently, it is more than likely that our WFA labeling does not reflect the full expression pattern of PNNs, so some caution is warranted in comparative interpretations of our study, which may be resolved through further studies in the mouse using these alternative PNN markers.

A second methodological caveat is our use of the VGAT-Venus mouse strain for identifying inhibitory neuronal cell types [36,37]. As with the above, the general utility of VGAT-Venus transgenic labeling for identifying inhibitory neuronal cell types relies on the general overlap of expression with gamma-amino butyric acid (GABA)-ergic cells. In this regard, we have demonstrated previously that VGAT-Venus cells overlap with over 90 percent of GABAergic neurons in the IC [26], with similar findings reported for the cerebral cortex [36]. Consequently then, it is likely that our quantification of PNN association with inhibitory neurons may be slightly underestimated. Moreover, this transgenic strain, like many transgenic strains, was raised on the C57BL6/J background, which has been noted to have early onset of age-related hearing loss [39,40], due to early deterioration of the stria vascularis resulting in early and progressive hair cell loss in the cochlea and that may in turn affect central inhibitory pathways, potentially confounding the present findings [48,49,50]. As such, we examined the expression of WFA-labeled PNNs at various ages, but did not find any statistically significant differences across the ages that we surveyed (Figure 2, Figure 5 and Figure 7). However, it remains to be determined whether the present findings are generally indicative of PNN formation in central auditory structures of other mouse strains.

In general though, our WFA-labeled PNN expression matches broadly those observed in related studies of the IC [11,12,14,15,35] and A1 [11,16,18,19,20] of other species. In the IC, we found the densest expression in the central nucleus, with lesser expression in the surrounding lateral nucleus and dorsal cortex (Figure 1 and Figure 2), which closely resembles the pattern observed in the guinea pig [12]. However, unlike the guinea pig, we found that most WFA-labeled PNNs were not associated with inhibitory neuronal cell types (Figure 1 and Figure 2), which could either reflect the methodological issues described above or species-specific differences [12]. In contrast, in A1, we found a robust association of WFA-labeled PNNs with inhibitory neuronal cells (Figure 6 and Figure 7), analogous to prior studies indicating an association with interneurons in the cortex, particularly parvalbumin-positive neurons [19,46]. Moreover, we found a regional clustering of WFA-labeled PNNs in the rostral regions of auditory cortex, which may reflect chemoarchitectonic areal boundaries, analogous to those described in the cat, gerbil and rat [18,20].

In addition, we found that WFA-labeled PNNs were densely, although not ubiquitously, expressed in the TRN (Figure 4 and Figure 5). As noted above, the TRN is a critical structure providing inhibition to the auditory thalamus in rodents, which lack auditory thalamic interneurons [28,29]. Although PNN expression revealed by WFA labeling was not evident in the MGB (Figure 3), we found intense expression of WFA-labeled PNNs in the putative TRN region innervating the auditory thalamus on the lateral extent of the TRN in our semi-horizontal sections (Figure 4E). The apparent absence of PNNs in the MGB, as noted above, may be a limitation of the method of detention via WFA labeling, rather than PNN specific antibodies [47]. However, not all inhibitory neurons in the TRN were surrounded by WFA-labeled PNNs, suggesting potentially unique information processing roles for those different types of TRN inhibitory neurons. Indeed, TRN inhibitory neuronal types might be distinguished in the same manner as a reported general association in the cortex of PNNs with neurons expressing the potassium channel subunit (Kv3.1b) [51]. Classifying the differences in intrinsic membrane properties of those TRN neurons associated with PNNs *vs.* those unassociated could elucidate the distinct network and synaptic properties inherent in the reticulothalamic circuitry [52].

The degree to which these PNNs affect the overall processing of auditory information in the mouse still remains to be determined, however their potential role in synaptic modulation and plasticity hint at possible functional roles, particularly in auditory learning [6,7,8,9]. Here, we have largely focused on PNN expression at relatively higher levels of the auditory pathways, although undoubtedly their expression in lower brainstem structures of the mouse is critical for auditory processing [5,17]. Although their function in auditory processing remains to be determined, the denser clustering of WFA-labeled PNNs in the central nucleus of the IC and layer 4 of the primary auditory cortex is consistent with the notion of these regions as the principal regions for the high-fidelity transfer of auditory information [28,33], and presumably these auditory regions are less inclined plastically [24,25,28,33].

## 5. Conclusions

We characterized the expression of perineuronal nets (PNNs) in the vesicular gamma-amino butyric acid transporter (VGAT)-Venus C57BL/6 transgenic mouse line in the inferior colliculus (IC), thalamic reticular nucleus (TRN) and the primary auditory cortex (A1). Each of these structures exhibits distinct patterns of PNN expression that likely support their individual roles in the processing of auditory information. It remains for future investigations to determine the nature of these PNN contributions and how pathological, developmental or age-related alterations to PNNs affect their normal functioning.
